# Assessment of Metals Concentrations in Soils of Abu Dhabi Emirate Using Pollution Indices and Multivariate Statistics

**DOI:** 10.3390/toxics9050095

**Published:** 2021-04-25

**Authors:** Yousef Nazzal, Alina Bărbulescu, Fares Howari, Ahmed A. Al-Taani, Jibran Iqbal, Cijo M. Xavier, Manish Sharma, Cristian Ștefan Dumitriu

**Affiliations:** 1 College of Natural Health and Health Sciences, Zayed University, Abu Dhabi P.O. Box 144534, United Arab Emirates; yousef.nazzal@zu.ac.ae (Y.N.); fares.howari@zu.ac.ae (F.H.); Ahmed.Al-Taani@zu.ac.ae (A.A.A.-T.); Jibran.Iqbal@zu.ac.ae (J.I.); cijo.xavier@zu.ac.ae (C.M.X.); manish.sharma@zu.ac.ae (M.S.); 2 Department of Civil Engineering, Transilvania University of Brașov, 5 Turnului Str., 900152 Brașov, Romania; 3 SC. Utilnavorep SA, 55 Aurel Vlaicu Bd., 900055 Constanța, Romania

**Keywords:** soil pollution, multivariate analysis, PCA, pollution indices

## Abstract

The aim of this study was twofold. Firstly, we performed a land capability class determination of the agricultural soils from the Abu Dhabi Emirate, the United Arab Emirates, based on the concentrations of 17 chemical elements determined in the soil samples collected from 84 locations. Secondly, we assess the soil pollution with different metals, using several pollution indices. The results of Principal Component Analysis (PCA) shows that four principal components (PCs) are responsible for describing the total metals concentrations’ variance, the highest contribution on PC1 being that of Mn, and Cr, on PC2 that of Fe, on PC3 that of Cu, and on PC4 that of Al. After determining the optimal number of clusters, we classified the sites into three clusters, while the studied metals were grouped function on their concentrations. Then, we used five indices to assess the pollution level of the soil at the study sites and in the clusters. The geo—accumulation index (I_geo_) indicates uncontamination/moderately contamination with Cu in cluster 1, uncontaminated/moderately contaminate soils with Cd, Cu, and Ni in cluster 2, and uncontaminated/moderately contaminated soil with Cu and moderately contaminated with Pb, Zn, and Ni in cluster 3. By comparison, the enrichment factors overestimate the pollution of the studied sites. The pollution load index (PLI) indicates a baseline level of pollution at 14 sites and the deterioration of the soil quality at four sites. The Nemerow pollution index provides similar results as PLI.

## 1. Introduction

Nowadays, anthropogenic activities lead to a biological and chemical imbalance in metals’ geochemical cycles, disturbance, and acceleration of metals’ natural geochemical process [1]. The atmospheric pollution and the deposition of suspended particles from the atmosphere contribute to the environmental quality deterioration [2].

Even if some elements are naturally found in soil, their accumulation above a certain level may be toxic for the ecosystems and human health [3].

Heavy metals are, by definition, metals with a density greater than 5 mg/cm^3^. This term is often used to name metals and metalloids (i.e., arsenic) associated with contamination and potential toxicity [4]. Since the heavy metal list is not well defined, in the following, we shall avoid its use, as recommended by the International Union of Pure and Applied Chemistry [5].

The natural and artificial stress due to various anthropogenic activities contributes to the metals’ accumulation [4,6,7,8]. Anthropogenic activities lead to a biological and chemical imbalance in metals’ geochemical cycles, disturbance. Moreover, the accumulation of high quantities of metals in soil is a risk factor for plants, animals, and human health [9,10,11,12,13,14]. On a closer look, the agricultural practices impact the soil’s nature (physical, chemical, mineralogical, and biological). Variations of the soil characteristics, which occur depending on the farming management practices and soil formation processes, significantly impact the agricultural products. Agriculture sustainability is the most critical factor in deciding the crops’ type [15]. Additionally, uncontrolled use of inorganic fertilizers, biosolids from slugged and manures, wastewater, pesticides, agrochemicals, and extensive irrigation schemes can also affect plant’s metabolic activities. Literature reveals that excessive anthropogenic actions at the regional and global levels play an essential role in pedogenesis [16,17].

High concentrations of metals, such as Cd, Cu, Zn, and Pb, are usually associated with the impacts of urbanization [18,19,20,21,22]. Increased concentration of Cr, Cu, Ni, and Zn results in phytotoxicity [23,24]. Cd and Pb are food-chain contaminants, although only Cd can readily enter the food chain by the plant uptake. In high quantities, Al, As, Co and Sr are very toxic as well. Fe availability depends on the soil’s pH. Mg, Na, K, and Ca are either primary or secondary soil macronutrients [25,26,27,28,29]. Mn is essential for photosynthesis and plant growth [27,30,31]. Barium naturally occurs on minerals in trace quantities. Present as free ionic metal, Ba2+, and other metallic elements can affect the plants’ development, from the subcellular to the ecosystem level [32].

Understanding the soil characteristics is necessary to interpret the soil system’s complexity [33]. Appropriate low amounts of metals are essential for animal and human health, while excessive amounts are harmful to all living beings—humans, plants, and animals [34]. Assessment of toxic elements in the soil is essential to determine the pollution level and reduce the contamination [35,36] to retrieve the full capability of the terrain. Additionally, knowing the spatial distribution of land cover types is necessary to assess the presence of different elements in the background and identify the contamination areas [37]. Based on this knowledge, lawmakers must implement new policies and procedures to avoid soil pollution.

Scientists studied pollution levels in the soil by a variety of methods, like testing statistical hypotheses [7], regression models [9], regional models [2], geostatistical analysis [17]. Multivariate statistical methods [11,12,38] provide a valuable tool for simplifying complex data sets by unifying the study of many variables into a single analysis. Unlike univariate or bivariate methods, this technique correlates large data sets to determine the relationships between variables. It overcomes difficulties linked to the multidimensionality, errors propagation, unaccounted data, and disparity due to experimental errors and noise. It helps to clarify the nature of the problems at hand and support their solving and potential applications. Multivariate methods use information from relationships among variables to improve the factors estimations and identify various causes of variations at different spatial scales [39,40]. The application of multivariate statistical techniques on the metals’ accumulation helps drawing conclusions based on big original data sets [41,42]. It provides a precise interpretation of the environmental circumstances and soil variables. Factors controlling the soil characteristics and their correlations can be determined using the correlation matrix, Principal Component Analysis (PCA), and Cluster analysis.

Literature survey reveals different multivariate GIS approaches for determining the heavy metal contamination in agricultural soil [8,43,44,45,46,47,48,49,50] in many parts of the world, but only a few articles are focusing on the analysis of the metals concentrations in soils [38], sediments [51,52,53] and water [54,55,56,57], in the United Arab Emirates. Apart from the mentioned articles [38,51,52,53,54,55,56,57], only one study [58] presents the soil characteristics in the Eastern Arabian Desert Region using as investigation tools the ICP-OES with GIS Mapping.

The objective of this work is twofold. The first goal is to classify the agricultural soils in the Abu Dhabi Emirate, considering the concentrations of 17 elements in soil samples collected from 84 locations and employing a multivariate statistical approach. The second goal is to determine the degree of agricultural soil contamination utilizing individual and complex pollution indices. We also investigate the relationship between the pollution degree and the sites’ appurtenance to the clusters determined at the first stage.

Therefore, the present study will lead to locating spatial distributions of the elements in soil, site-specific pollution characteristics, and a summative indication of the elements grouping. These results are useful for a proper understanding of soil quality, draw significant conclusions on the topic of interest for the soil scientists [48,59,60], and promote a well-balanced agricultural use of areas in the future.

## 2. Material and Methods

### 2.1. Study Area

**Figure 1:**
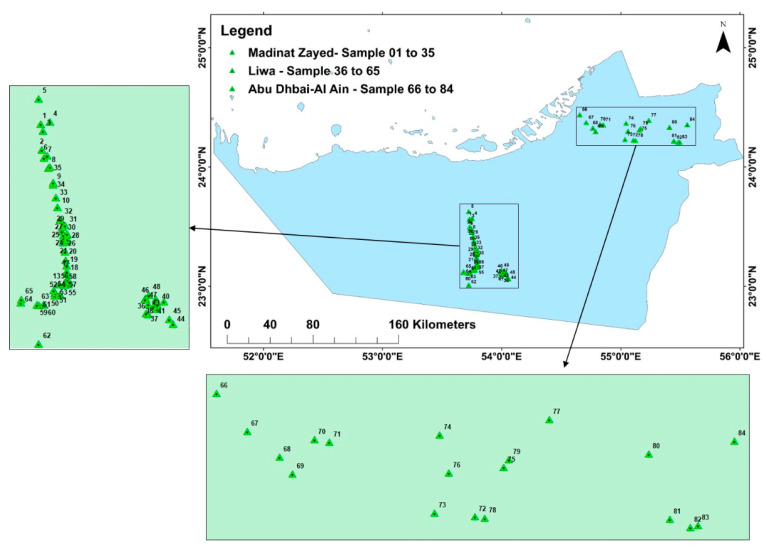
Study area and the sampling locations.

Abu Dhabi Emirate, the largest Emirate of the United Arab Emirates, is situated along the Arabian Gulf, between 22.5°–25° north latitudes, and 51°–55° east longitudes (Figure 1). It is composed of three geologic units (1) a coastal region of tidal flats, Sabkhas, and terraces along the Arabian Gulf, (2) the piedmont plain near Al-Ain city, and (3) the internal dune region covering the most area of the Emirate [61].

Geologically, Abu Dhabi shares the northeastern corner of the Arabian platform with Oman, remained relatively stable for millions of years. This Emirate resided in a tropical and subtropical climate since the Paleozoic end when it experienced various climate changes. Sediments, predominantly eolian dunes, of the Holocene and Pleistocene age dominate the surficial geology. Remnants of the Hajar Mountains are predominant in the eastern parts of the Emirate displaying the only natural hard rock outcrops that appear in this Emirate. The salt diapirs of the Hormuz Formation seen at Jabal Az Zannah and many offshore islands (e.g., Sir Bani Yas) are the oldest formations. These diapirs represent Cambrian materials, extruded through fissures in materials due to the overlying rock weight. Other minor occurrences of pre-Permian materials exist on the eastern borders of the Emirate to the east of Jabal Hafit. Limestone and marls of Tertiary age compressed and folded in the formation of the Hajar Mountains from Jabal Hafit. The rest of the Emirate territory contains extensive sand and gravel plain with a thick blanket of eolian dunes formed by the prevailing wind (Supplementary Material, Figure S1) [62]. 

Broader soil categories in the Abu Dhabi Emirate are sandy, sandy calcareous, gypsiferous, saline, saline gypsiferous, and hardpan soils. Sandy desert soils (Entisols) are dominant, followed by Aridisols to a relatively lesser extent, and the Inceptisols [63] (Supplementary Material, Figure S2).

Abu Dhabi Emirate’s land use includes urban, farming, rangeland, oil fields, cemeteries, commercial facilities, mangroves, quarries, and water reservoirs area [64] (Supplementary Material, Figure S3).

### 2.2. Sample Selection

For this study, we collected soil samples from 84 different agricultural lands in the Abu Dhabi Emirate using the following procedure. For each agricultural land, we designed a zig-zag pattern and collected 15 samples [65]. Before the samples’ collection, the surface litters and a thin surface of the soil (about 5 cm) was removed for discarding the surface debris (such as plant residues, mulch, or turf thatch) from the ground.

Then, we cut up to about 20 cm (from the surface) depth of the soil slice using a hoe. We put in plastic buckets equal quantities of the soil samples from each sampling point and mixed them thoroughly. Nearly 1 kg of this mix was transferred to a plastic bag and labeled. Samples were moved to a temperature-controlled cooler box, maintained the temperature between 25–35 °C, and transferred the same day to the laboratory for further analysis.

### 2.3. Geochemical Analyses

We dried the samples for 24 h using a hot air oven at 60 °C. Then, we crushed them using mortar and pestle and passed them through a 63 µm sieve. Crushing and sieving were performed to homogenize the samples, to reduce analytical variability. The analysis was carried out according to the current U.S.EPA 3051A [66] guidelines with triplicate sample preparation. About 0.5 g of each homogenized sample was weighed accurately using a semi-micro balance (Ohaus) and collected in PTFE digestion vessels. Samples were digested using 9 mL of Nitric acid (Sigma Aldrich) and 3 mL of hydrochloric acid (Sigma Aldrich). The rapid addition of thermal energy to the reactive mixture is highly hazardous. To avoid this uncontrollable reaction, samples were predigested in a hood with loosely capped vessels that allow the exit of dangerous gases in a fume hood. Further digestion was carried out in a microwave digestion system (MARS-6, CEM Corporation) with the temperature program of 175 ± 5 °C in approximately 5.5 ± 0.25 min and remained at 175 ± 5 °C for 4.5 min. We also prepared reagent blanks to check for background contamination without the addition of soil samples. Digested samples were diluted further with ASTM Type-1 water (Millipore), filtered using 0.45 µm PTFE filter (Whatman). The resulting solution was analyzed in an Inductively Coupled Plasma—Optical Emission Spectrometer (ICP-OES, PerkinElmer Avio 200).

We followed a strict quality control (QC) procedure throughout the analysis. The method accuracy was verified using a spiked Certified Reference Material ERM^®^-CC141 [67]. We verified the metal’s recovery at the beginning of the analysis and after every tenth sample. Recovery ranged from 86% to 108%. For estimating the precision, we computed the mean values from the triplicate analysis for each study metal. The elements analyzed and their absorption wavelength are the following Al (396.153), As (193.696), Ba (233.527), Ca (317.933), Cd (228.802), Co (228.616), Cr (267.716), Cu (327.393), Fe (238.204), K (766.490), Mg (285.213), Mn (257.610), Na (589.592), Ni (231.604), Pb (220.353), Sr (407.771), and Zn (206.200).

### 2.4. Multivariate Analyses

Firstly, we computed the basic statistics of each concentration series and compared them with the results from the literature.

We built the correlation matrices of the metals’ concentrations series and computed the significance levels for assessing the elements’ correlations in the soils. 

Principal Component Analysis (PCA) [68] is employed to extract the main components and identify the pollutants’ sources. For selecting the principal components (PCs), three approaches are generally used: the Kaiser Criterion [69], the variance explained criteria (percentage of variance), and the Scree plot [70]. Based on the Kaiser Criterion, the selected PCs are those with eigenvalues greater than 1. The Scree plot and the variance explained criteria are utilized together with the Kaiser criterion since sometimes the last one overestimates the number of components to be retained [71,72]. A Scree plot displays the eigenvalues in a downward curve, ordering them from largest to smallest. The “elbow” of the graph where the eigenvalues seem to level off is found, and the PCs to the left of this point are considered significant. Based on the variance explained criterion, the retained PCs should explain at least 70–80% variance. 

The next step is to group the sites based on the elements’ concentrations in the soils’ samples. For this aim, we use divisive hierarchical clustering. Compared to the agglomerative clustering (that could be employed for the same purpose), the divisive hierarchical clustering is more efficient (is computationally less expensive) and more accurate, considering the data distribution for partitioning decisions [73]. For performing the clustering, the variables were standardized. 

We used the R software for performing all the statistical analyses and modeling.

### 2.5. Evaluating the Soil Pollution by Pollution Indices

In the last part of the study, we compute different indices for assessing the soil pollution level by Cd, Cu, Ni, Pb, Zn, Cr, Co, Mn, As, Ba, called heavy metals in different studies [74,75]. They are the geo-accumulation index (I_geo_), single pollution index of a certain metal (PI), the enrichment factor (EC), and the Pollution Load Index (PLI), and the Nemerow index.

For the metal *i*, I_geo_ is calculated using the formula [74,75,76]:

I_geo_ = log_2_ (*C_i_*/1.5*B_i_*), (1)

where *C_i_* is the measured concentration of metal *i* in the soil, 1.5 is a factor used to minimize possible variations in the background value of metal *i*, and *B_i_* is the background value for the studied metal.

The reference values of Muller [76] and the associated contamination levels are presented in Supplementary Material, Table S1.

The single pollution index of a metal *i* (*PI*) is defined by [74]:

PI = *C_i_* /*B_i_*. (2)

Function of the PI values, the classes of contamination are: uncontaminated (PI < 1), low (1 < PI < 2), moderate (2 < PI < 3), strong (3 < PI < 5), and very strong (PI > 5) [74]. 

The enrichment factor (EF) [74,75,77]

EF = [*C_i_*/*LV* sample]/[*GB*/*LV* background], (3)

where [*C_i_/LV* sample] is the ratio of the content of analysed heavy metal, and that of a selected metal (Al, Ca, and Fe in this study) in the sample, and [*GB*/*LV* background] is the ratio between the content of analysed metal and that of a selected metal in the background.

Scientists established the following enrichment categories: deficiency to minimal (EF < 2), moderate (2 < EF < 5), significant (5 < EF < 20), very high (20 < EF < 40), and extremely high (EF > 40) [74,77]. If the values of EF are in the interval 0.5–1.5, then the content of the study metal results from natural processes [75].

Pollution Load Index (PLI) is usually used to assess the contamination extent by metals in soils. Its computation is done by the formula [78,79,80,81,82]:

PLI = [*PI*_1_ • *PI*_2_• … • *PI_n_*]^1/*n*^ (4)

where *PI_i_* (*i* = 1, … *n*) is the single pollution index of the metal *i*, and *n* is the number of metals. 

The Nemerow Pollution Index (*PI_Nemerow_*) is computed by [83,84]:

*PI_Nemerow_* = [1/2(*PI_M_*^2^ + *PI_max_*^2^)] (5)

where *PI_M_* is the average of the pollution indices taken into account and *PI_max_* is the maximum value of these indices. Values under 0.7 indicates clean environment, between 0.7 and 1—warning, in the interval 1—2—slight pollution, between 2 and 3—moderate pollution and higher than 3—heavy pollution.

## 3. Results and Discussion

### 3.1. Geochemical Setup

Table 1 displays the basic statistics—minimum and maximum concentration, mean values (mg/kg), standard deviations, skewness, and kurtosis coefficients—for all elements’ concentrations in the study samples.

Cd’s average concentration in the soil samples is higher than the average crustal abundances and average world soil concentrations (Table S2, Supplementary Material) [77,85,86,87]. The average concentrations of Cu, Ni, Zn, Mg are higher than the average world soil concentrations but lower than the average crustal abundances. The As average concentration in the samples is at least 700 times lower than the reference concentrations (Table S2, Supplementary Material). The rest of the elements have average concentrations lower than the reference limits [77,85,86,87].

The high standard deviations of some concentration series (Fe, Al, Ca, Mg, Na, and K), together with the high skewness coefficient (for Pb, Zn, Co, Ba, Mn, Na, K), indicate inhomogeneous distributions of these elements in the soil at the studied sites. The skewness values of Cd, Cu, Ni, Pb, Zn, Co, Ba, Ca, Mg, Sr indicate positively-skew concentrations series. The high deviation from a Gaussian distribution is emphasized by the kurtosis coefficients, which are very high for Cd, Cu, Pb, Zn, Co, Ba, Mg, Sr (indicating leptokurtic distributions). Therefore, at first sight, the elements that might provoke soil pollution at the study sites are Cd, Cu, Ni, Zn, and Mg.

Cadmium is a heavy metal that exists naturally in the soil with a concentration range between 0.03–0.15 mg/kg and is one of the most toxic metals [88]. Cd concentrations in the analyzed samples range from 0.026 to 1.491 mg/kg (Table 1).

Cd is a non-biodegradable element, so its transfer to the human food chain should be avoided. Long-term exposure to cadmium, including contaminated food ingestion, leads to cancer and organ system toxicity such as skeletal, urinary, reproductive, cardiovascular, central and peripheral nervous, and respiratory systems. Cadmium is used as alloys in electroplating (auto industries), in pigments and rechargeable Ni-Cd batteries production, detergents industry, and refined petroleum products [29,58,89].

Samples collected from the agricultural lands situated in the neighborhood of the Mussafah industrial area and neighboring petroleum stations have Cd concentrations two-three times higher than those determined by Turekian and Wedepohl [78], Tayler [85], and Mason [86]. Since fertilization increases the risk of Cd transfer to the food chain, and the Cd content in all the samples is high, it is necessary to monitor the pollution sources, especially the agricultural use of phosphate fertilizers, for avoiding the augmentation of the Cd concentration above a warning limit. 

Copper is considered naturally as a micronutrient for healthy soils and plant growth [90]. Usually, it occurs in a limited amount in sandy soils. Copper enters the atmosphere, mainly through the release during fossil fuel combustion. The wind transports it at distances from the production point and the rain facilitates its deposition on the soil. Phosphate production, industrial settings, landfills, and waste disposals increase the pollution with copper [91,92]. Ingestion of contaminated food causes anemia, digestive system irritation, liver and kidney damage [58,92,93].

Researches [86,87] show that the average concentration of Cu in the soil is about 20 mg/kg. The analyzed samples’ concentrations in the interval 0.892–158.672 mg/kg (Table 1). The concentrations of the samples collected at the sites situated near the Mussafah area (Abu Dhabi—the first 35 samples) were more than two times higher than 20 kg/mg. Samples 75 (158.67 mg/kg), 68 (87.60 mg/kg), and 67 (81.64 mg/kg), collected from locations situated at less than 100 m distance from the highway, had the highest concentration. 

The Ni average crustal abundance is about 75 mg/kg [74,85,86]. Nickel’s source could be both natural and anthropogenic. The major anthropogenic sources of soil pollution with nickel are metal plating and electroplating industries, fossil fuels combustion, and nickel mining [59]. Ni is released into the air by power plants and trash incinerators and settles to the ground after undergoing precipitation reactions. Ni can accumulate in plants by uptake from contaminated soils [3,6,7,8,9,10]. Since Ni is very toxic in high concentrations, causing harmful effects to the human immune and reproductive system [58], periodic screening is needed to ensure Ni concentrations within acceptable limits.

Ni’s concentrations in the collected samples ranged between 1.424 and 416.381 mg/kg (Table 1). The upper limit is more than five times higher than the crustal average, proving the human activity impact. The highest concentrations is noticed in the samples collected from the Al Ain region (416.381 mg/kg—site 68, 385.93 mg/kg—site 76, 366.96 mg/kg—site 67, in zones situated close to the roads).

Zinc is a common element in the earth’s crust, with an average crustal abundance of 75 mg/kg, and an average concentration in soil of 50 mg/kg (Table 1). Zn is an essential element for normal crops’ growth. For agricultural soils with Zn deficiency, it is used as fertilizer (in small quantities) [94]. In high amounts, Zn becomes toxic, especially if the soil is acidified to increase other nutrients’ concentrations on a continuous fertilization process applied for extended periods [58,95].

Zn’s concentration in the analyzed soil samples ranges between 1.867 and 376.479 mg/kg. The samples collected from the agricultural fields in the Liwa area had a concentration under 17 mg/Kg, while the samples collected from the Al Ain area presented very high concentrations (the highest was recorded at site 68–376.479 mg/kg, followed by 224.74 mg/kg, registered at site 72). Zn and Cd may also accumulate in soils adjacent to roads, form sources as tires, lubricant oils, and waste combustion.

The Pb concentrations in the samples collected from the Abu Dhabi region are under 1.071 m/Kg, showing the absence of contamination. Only two samples from the Liwa zone have concentrations above 10 mg/kg (13.84 and 18.07 mg/kg). The highest concentration of Pb were found in the samples 68 (113.71 mg/Kg) and 71 (104.20 mg/Kg), collected in the Al Ain zone. The lead pollution in this region could come from waste incinerators, lead-acid battery manufacturers and piston-engine aircraft operating on leaded aviation fuel facilities in the sampling sites’ neighborhood.

The Cr content is under the literature value (100 mg/kg) in all but five samples. The concentrations of As, Co, Na, Sr and K are tiny compared to those from literature, those of Ba are above 500 mg/Kg (found in the literature as reference limit) only in one sample. Mn concentration is 1.61 to 75.16 times lower than the average value found in the soil samples all over the world [86,87], showing a deficiency of this element in soil.

The calcium content is above the crustal abundance in soils from the Al Ain and Liwa regions (samples 36–84) and at least five times lower than the soil average in the other samples due to the soil lithology.

Aluminum is the third most abundant element found in the earth’s crust and may be toxic for human beings, animals, and plants [95]. In the study samples, the Al content is at least 1.6 times under the average soil concentration, so there is no contamination risk. No pollution risk presents Fe in the amount found in the samples.

Magnesium is the eighth-most abundant element found in the earth’s crust (20,900 mg/kg) and the third in seawater. It is considered that this element has low toxicity and is not dangerous for the health [92]. Mg concentrations in the study samples are 7.26 to 39.29 times higher than the worldwide average (500 mg/kg). Studies show that the major source of base Mg cations in the air is dust from soil erosion, emissions from urban and industrial sources, and fuel combustion [96,97,98]. In the study region, the origin is mainly the lithology.

### 3.2. Multivariate Geostatistics

Firstly, the correlation matrix of the studied elements has been built. Figure S4 in Supplementary Material provides the matrix containing the p-values corresponding to the correlation coefficients between the metals concentrations in the analyzed soil samples from the Abu Dhabi Emirate. It results that: (a) Pb is highly positively correlated with Ni, Ba, and Zn; (b) Ni is highly positively correlated with Pb, Zn, Cr, Co, Ba, Mn, and Ca; (c) Cr is highly positively correlated with Ca; (d) Mn is highly positively correlated with Ni, Zn, Cr, As, Ca, and K. A high positive correlation among metals possibly suggests a common origin, like traffic flow or industrial activity in the study area. 

After performing the Bartlett sphericity test (at the significance level of 5%), it resulted that data reduction could compress the available data meaningfully (because of the *p*-value < 0.05). Therefore, we utilized three criteria for assessing the number of principal components (PCs). Scree plot is presented in Supplementary Material, Figure S5.

The plotting of eigenvalues (Oy-axis) against the PCs (Ox-axis) provides insight into the maximum number of components that should be extracted. There are four eigenvalues greater than 1 (6.873, 2.789, 1.610, and 1.246), explaining 40.431% and 16.406%, 9.470%, and 7.328%, respectively of the total variance (Supplementary Material, Table S3). The percentage of the total variance explained by the first four PCs is 73.634%. Therefore, we retained only the first four PCs.

**Figure 2:**
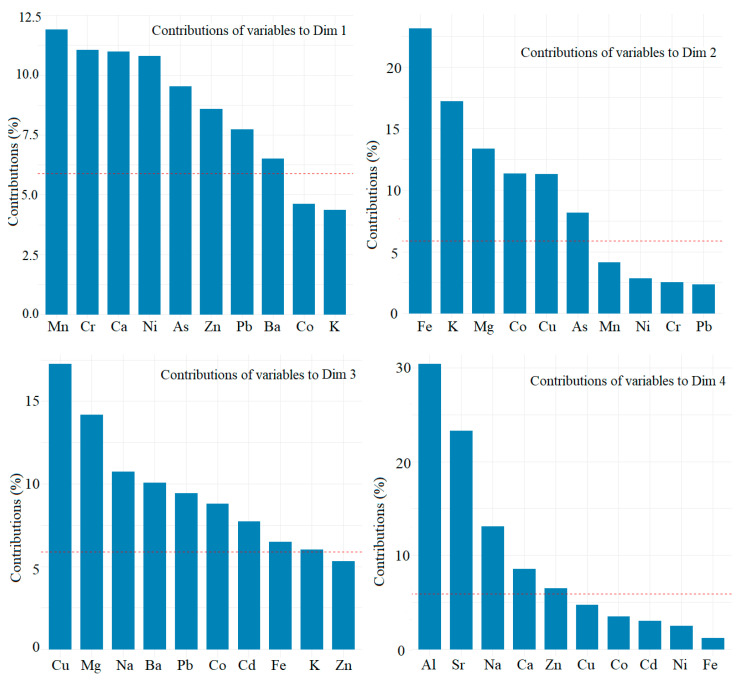
Contributions of the elements on the first four PCs.

The highest ten contributions on the first four components of the elements are shown in Figure 2 and Table S4 (Supplementary Material).

The elements with the highest contributions on the first PC are Mn (about 11%), Cr, Ca, Ni, As, Zn, Pb, and Ba. The elements with the highest contributions on the second PC are Fe (about 25%), K, Mg, Co, Cu, and As. The highest contributions on the third PC are those of Cu (about 17.5), Mg, Na, Ba, Pb, Co, Cd, and Fe, whereas the highest contributions to the fourth component are those of Al (about 30%), Sr, Na, Ca, Zn.

The rotated factor loading matrix (Supplementary Material Table S5) shows that the highest loadings (over 0.8) on the first component are those of Ni, Zn, Pb, on the second one, those of Mg, and Co, and one the third one those of K, and As. On PC4, the highest loading corresponds to Al (0.773) and Sr. 

The highest loadings on PC1 suggest that the elements’ sources are emissions from the traffic flow and industrial activities from zones situated in the study sites’ neighborhood. The sources of the components with the highest contributions on PC2 seem to be lithology and industry.

**Figure 3:**
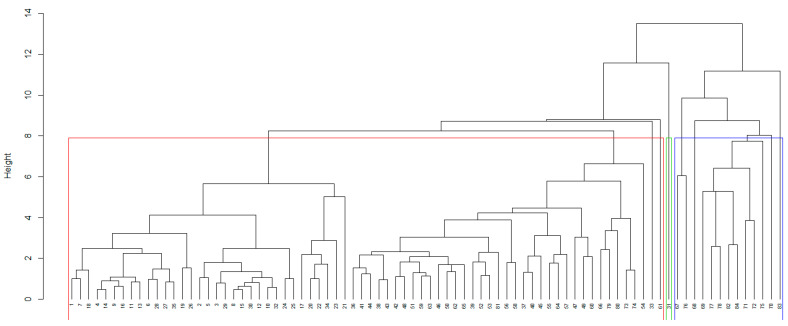
Dendrogram for the sites grouping.

**Figure 4:**
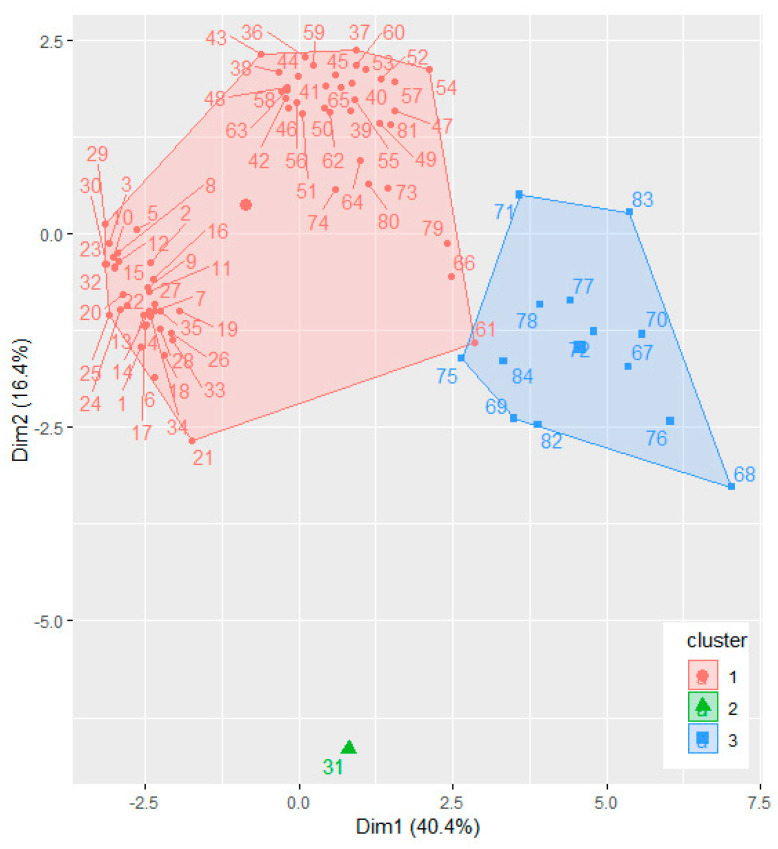
The cluster plot for the sites grouping.

Using 30 methods implemented in the NbClust package in R, the optimum number of clusters for the sampling sites grouping (based on the concentrations of the elements in the soil samples) was determined to be 3. The dendrogram (Figure 3) and the cluster plot (Figure 4) present this classification.

Figure 4 shows good separation of the clusters. The second cluster contains only site 31 since the concentrations recorded for different elements significantly different from those situated in the other clusters. For example, the concentrations (mg/kg) of Ni (61.473), Zn (11.222), Cr (14.202), Co (3.556), Ba (9.580) are much lower than those recorded for the samples from Cluster 3, whereas the concentrations of Fe (4632.881), Mn (19.634), Al (2156.231) are higher than those recorded for the samples in Cluster 1.

**Figure 5:**
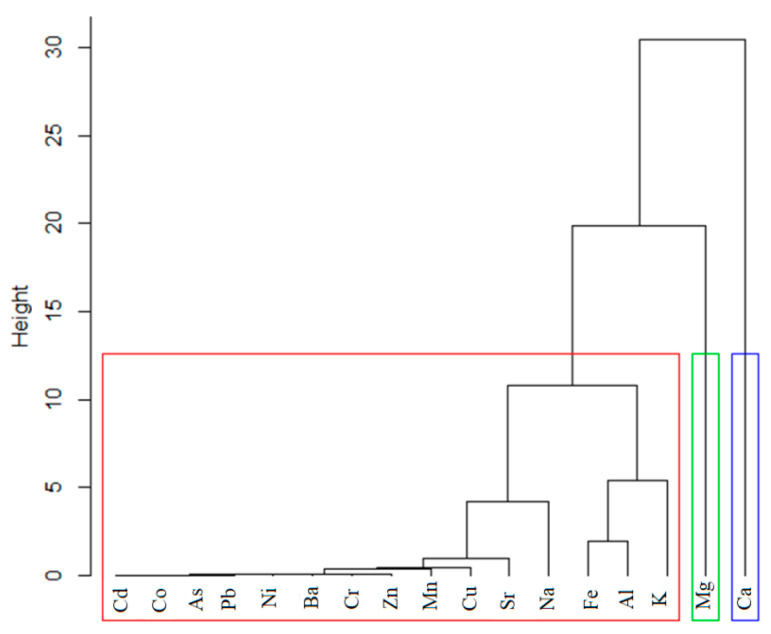
Dendrogram for the grouping of the elements.

Figure 5 shows the clustering of the chemical elements in soils using a dendrogram. Ca and Mg are situated in different clusters, whereas all the other elements belong to another cluster. Going deeper, in the cluster marked with the red border, Fe, Al and K have similar concentrations. Taking into account the rule that the smaller the lines connecting the elements are, the smaller the dissimilarities are Cd, Cu, Ni, Pb, Zn, Cr, Co, Ba, As, and Mn are in the same group (the left-hand side of the dendrogram from Figure 5).

Therefore, in the next stage of this study (taking into account the results from Section 3.1), we computed different indicators for assessing the soil pollution with these elements. 

Table 2 contains the I_geo_ for the average concentrations of all the elements and the clusters’ elements. It results that the soils are uncontaminated/moderately contaminated with Cu and Ni. The soils from cluster 1 are uncontaminated/moderately contaminated with Cu, those from cluster 2 are uncontaminated/moderately contaminate with Cd, Cu, and Ni. The soils from cluster 3 are uncontaminated/moderately contaminated with Cu and moderately contaminated with Pb, Zn, and Ni.

The I_geo_ computed for each site shows the following level of contamination:

- uncontaminated/moderately contamination with Cd at sites 33 and 76;
- uncontaminated/moderately contamination with Cu at sites 1–5, 7–16, 20–23, 26, 27, 29–35, 57, 66, 69, 80 and moderately contaminated soils at sites 6, 17, 24, 25, 28, 72 and 75;
- uncontaminated/moderately contamination with Ni at sites 31, 73, 74, 80, 81; moderated contamination at site 66, 69–71, 82–84; moderated to high pollution at 67, 68, 72, 75–79, and 84;
- uncontaminated/moderately contamination with Pb at sites 64, 66, 67, 69,71, 72,75, 78 and moderately contamination at 68, 70, 76, 77, 82, and 83;
- uncontaminated/moderately contamination with Zn at sites 50, 52, 57, 67, 71, 75–78, 82, 83; moderately contamination at 68–70.
- The sites not mentioned are not polluted.

The highest contamination with Cu is found in the soil from clusters 1 and 2, whereas the highest contamination with Ni, Pb, and Zn is found in cluster 3. Figure S6 from the Supplementary Material presents the chart of I_geo_ for the soil samples from cluster 3.

The computation of the single pollution index (PI) gave the following results:

- For Cd: 1 < PI < 2 (low soil pollution) at sites 35–55, 59–68, 70–73, 77,78, 80–84 and PI = 2.50 (moderate soil pollution) at 76;
- For Cu: low soil pollution at 2, 3, 5, 7, 9, 14, 16, 20, 26, 29, 31, 34, 64, 66, 69, 70, 76–79, 82; moderate soil pollution at 1, 4, 8, 10–13, 15, 21–23, 30, 32, 33, 35, 80; strong soil pollution at 6, 17, 24, 25, 27, 28, 67, 68, 72; strong soil pollution at 75;
- For Ni: low soil pollution at sites 31, 48, 49, 52, 55–57, 59, 60, 62, 65, 73, 74; strong and very strong soil pollution for the sites from cluster 3;
- For Pb: low soil pollution at sites 52, 64, 66, 71, 78, 81, 84; moderate at 67, 72, 75; strong soil pollution at sites 76, 77, 82, 83; very high soil pollution at 68, and 70;
- For Zn: low soil pollution at sites 39, 40, 42, 47, 49, 50, 52, 53, 62, 64, 66, 79, 82–84; moderate at 57, 67, 71, 75–78; strong soil pollution at 69, 70, 72; very strong soil pollution at 54, 68;
- For Cr: low soil pollution at sites 37, 40, 67, 68, 76.

Comparison of I_geo_ and PI show a higher degree of contamination and territorial extent when using the second index. The sites that appear polluted when using the I_geo_ are found among the sites classified as polluted when using the second index, generally with a higher pollution degree.

The average PIs for all the samples are 1.14 (Cd), 1.67 (Cu), 1.72(Ni), 0.90(Pb), 1.05(Zn), 0.43 (Cr), 0.02 (Co), 0.33(Ba), 0.24 (Mn), 0.00 (As), showing low pollution with Cd, Cu, and Zn. For the samples in cluster 3, the average PIs are higher—1.29 (Cd), 2.35 (Cu), 6.86(Ni), 4.01(Pb), 3.10(Zn), 0.88 (Cr), 0.07 (Co), 0.38(Ba), 0.50 (Mn), 0.00 (As)—by comparison with the values recorded for the cluster 2 (where only two PIs are above 1–1.57—Cu and 1.54 –Ni) or cluster 1 where only two PIs are above 1–1.11—Cd and 1.55—Cu).

Remark the strong average pollution with Cu, Pb, and Zn, and very high with Ni in cluster 3. This indicates the common origin of these pollutants, spread from the neighboring industrial sites or resulted from the traffic, as presented in Section 3.1. This result is in concordance with the finding from [38,52,53].

The enrichment factors have been computed using Al, Ca, and Fe as selected metals in Formula (3).

The average enrichment factors with respect to Ca, EF_Ca_, show significant enrichments with Cd (5.93) and Cu (12.18) due to significant enrichment with Cd, high and extremely high enrichment with Cu recorded at the first 35 sites in cluster 1 (situated in the Abu Dhabi area).

The average enrichment factors with respect to Al, EF_Al_ show moderate average enrichment with Cd, Ni, Pb, and Zn, significant enrichment with Cu. All the samples present a moderate enrichment with Cd. Significant and very high enrichments with Cu are notice for all samples but 1–35. Significant and very high enrichments with Ni are associated with all samples from cluster 3. Moderate and significant enrichment with Pb and Zn are determined in all samples from cluster 3.

The average enrichment factors with respect to Al, EF_Fe_ show significant enrichments with Cd (7.25), Cu (7.66), Ni(8.08), Zn(6.72), and moderate enrichment with Cr(2.98).

The computed enrichment factors (EF) with respect to Ca, Al, and Fe gave contradictory results: for Cd—significant, moderate, significant; for Cu—significant, significant, moderate; for Cr—deficiency, deficiency, moderate; for Ni—deficiency, significant, moderate; for Zn—deficiency, moderate, significant; for Pb—deficiency, moderate, moderate. These results may be explained based on the relative ratio of the samples’ elements, so the enrichment factors should be used together with other pollution indices for assessing the soil contamination [74]. 

Since there are significant differences beween the EF computed with respect to different metals, we recommend using EF together with other pollution indicators, EF overestimating the pollution.

**Figure 6:**
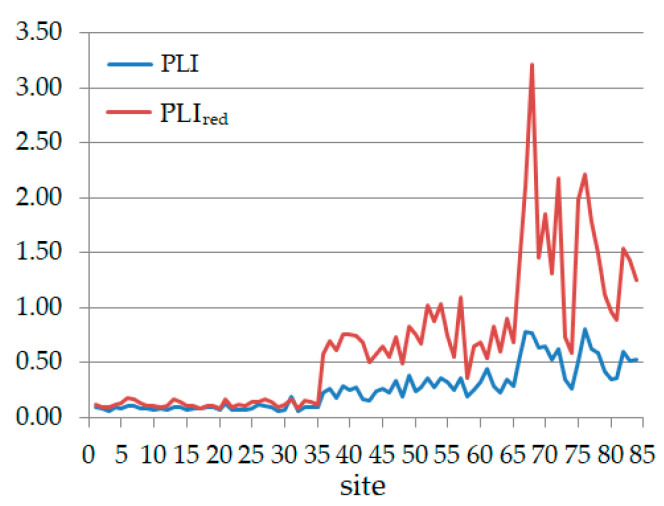
PLI and PLI_red_.

PLI values are from 0.0–0.83, but these are not relevant since there are very low values of individual PI for As, Ba, Co that contribute to diminishing the index values. Therefore, computing the PLI without the PI for the mentioned elements and denoting the new PLI by PLIred, we found that PLI < PLI_red_. PLI and PLI_red_ are represented in Figure 6. We remark significant differences between PLI and PLIred. If the values computed for sites 1–51 remain under 1 (showing perfection), the values for sites 52, 54, 57, 66–72, 75–79, and 82–84 are above this limit, showing baseline level of pollution or deterioration of the soil quality (for 66, 67, 72, 76).

**Figure 7:**
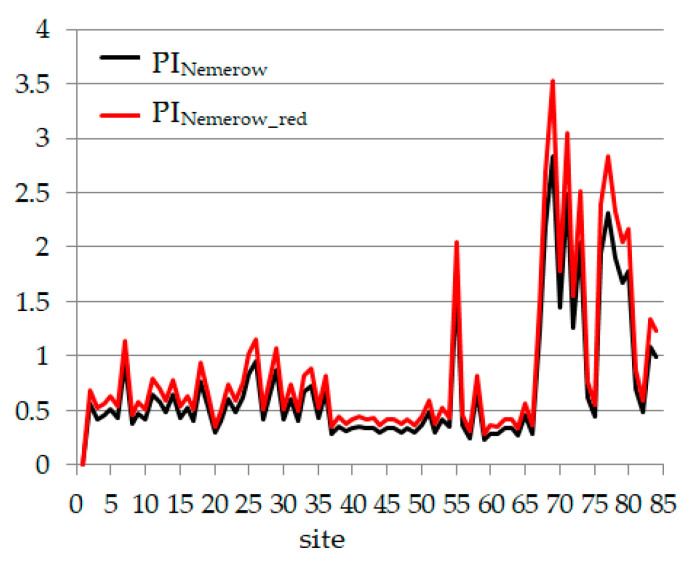
PI_Nemerow_ and PI_Nemerow_red_.

We performed similar computation as for PLI and PLI_red_ to obtain PI_Nemerow_ and PI_Nemerow_red_. Their values are presented in Figure 7. The sites from cluster 2 and most of those from cluster 1 indicate a clean environment or a warning level. The exceptions are samples 6, 24, 25, 28, 54 (slight pollution), 66 and 79 (moderately polluted sites). The samples from cluster 1 are mostly moderately polluted. These results are generally concordant with those provided by PLI and PLI_red_.

## 4. Conclusions

In this study, we proposed a multivariate analysis combined with pollution indices computation approach to addressed two objectives. The first one was evaluating the concentration of the 17 elements in agricultural soils from the Abu Dhabi Emirate and grouping the soils in clusters. The second one was investigating the agricultural soil pollution with different elements (using five pollution indices) and observing if the pollution extent is similar for the soil from the same cluster.

All indices show the highest contamination (especially with Cu, Pb, Zn, and Ni) in the soils included in the second cluster, indicating a common origin of pollutants in neighboring zones of the highways, roads with high traffic, emissions from fossil fuel, or industrial zones.

Enrichment factor analysis indicates that Cd, Ni, Zn, and Cr were highly enriched in soils, and they could originate from non-crustal sources. Thus, the enrichment factors should be used together with other pollution indices for assessing soil contamination. 

In the future, we intend to investigate this relationship for a better understanding of the connection between the multivariate analysis and the pollution indicators that could be successfully utilized together for assessing the soil pollution in different regions and taking documented measures for reducing the contamination or avoiding it.

## Figures and Tables

**Table 1:** Basic statistics of metal levels (mg/kg) for the collected soil samples.

Element	Min	Max	Mean	Standard Deviation	Skewness	Kurtosis
Cd	0.026	1.491	0.568	0.186	1.12	9.728
Cu	0.892	158.672	33.479	24.879	1.93	6.552
Ni	1.424	416.381	68.795	103.205	1.936	2.771
Pb	0.017	113.574	8.966	18.493	4.148	19.783
Zn	1.867	376.479	52.434	68.727	2.736	9.281
Cr	1.630	138.966	42.577	36.315	0.557	−0.585
Co	0.003	3.556	0.373	0.572	3.239	12.775
Ba	2.141	512.905	46.555	82.400	3.693	15.76
Fe	48.828	4632.881	1244.609	869.188	1.443	2.989
Mn	11.309	529.245	203.476	167.196	0.092	−1.454
As	0.001	0.011	0.007	0.003	−0.463	−1.548
Al	382.085	3401.256	1638.612	663.136	0.557	0.143
Ca	382.085	92,233.720	29,419.223	33,473.114	1.522	3.51
Mg	375.989	19,643.617	3932.598	3059.733	2.587	9.169
Na	17.830	9026.637	908.503	1377.381	3.449	15.1
Sr	7.090	1216.286	151.852	148.852	4.752	31.552
K	754.013	3582.475	2038.429	851.492	0.236	−1.521

**Table 2:** Geo—accumulation index of the average concentration of elements in soils (I_geo_). and contamination levels for selected metals.

Metal	I_geo_	Contamination Level (Cluster 1)	Contamination Level (Cluster 2)	Contamination Level (Cluster 3)
Cd	−0.40	−0.43	**0.06**	−0.22
Cu	**0.16**	**0.05**	**0.07**	**0.65**
Ni	**0.20**	−0.95	**0.03**	**2.19**
Pb	−0.74	−2.20	−9.79	**1.42**
Zn	−0.52	−1.16	−2.72	**1.05**
Cr	−1.82	−2.13	−3.40	−0.77
Co	−5.92	−6.56	−2.66	−4.35
Ba	−4.01	−5.19	−6.29	−1.99
As	−10.10	−10.22	−10.87	−9.58
Mn	−2.65	−2.97	−6.02	−1.58

## Data Availability

Data will be available on request.

## Supplementary Materials

The following are available online at https://www.mdpi.com/article/10.3390/toxics9050095/s1, Figure S1: The geological map of the study area, Figure S2: The soils map of the study area, Figure S3: Land use map of the study area, Figure S4: *p*-values corresponding to the correlation coefficients between the metals concentration, Figure S5: The scree plot, Figure S6: Igeo for the sites from cluster 3, Table S1: Geo-accumulation index (Igeo) for contamination levels, adapted from Muller, Table S2: Average crustal abundance and average world soils concentrations, Table S3: Results of the Principal Component Analysis—factors’ importance, Table S4: Principal components, Table S5: Rotated factor loading matrix.

## References

[1] D’Amore J.J., Al-Abed S.R., Scheckel K.G., Ryan J.A. (2005). Methods for Speciation of Metals in Soils. J. Environ. Qual..

[2] Bărbulescu A., Postolache F. (2021). New approaches for modeling the regional pollution in Europe. Sci. Total Environ..

[3] Barbeş L., Bărbulescu A., Stanciu G. (2020). Statistical analysis of mineral elements content in different melliferous plants from the Dobrogea region, Romania. Rom. Rep. Phys..

[4] Pourret O. (2018). On the Necessity of Banning the Term “Heavy Metal” from the Scientific Literature. Sustainability.

[5] Duffus J.H. (2002). “Heavy metals” a meaningless term? (IUPAC Technical Report). Pure Appl. Chem..

[6] Barbeş L., Bărbulescu A. (2017). Monitoring and statistical assessement of heavy metals in soil and leaves of *Populus nigra* L.. Environ. Eng. Manag. J..

[7] Barbeş L., Bărbulescu A., Rădulescu C., Stihi C. (2014). Determination of heavy metals in leaves and bark of *Populus nigra* L.. Rom. Rep. Phys..

[8] Jia L., Wang W., Li Y., Yang L. (2010). Heavy Metals in Soil and Crops of an Intensively Farmed Area: A Case Study in Yucheng City, Shandong Province, China. Int. J. Environ. Res. Public Health.

[9] Rădulescu C., Stihi C., Barbeş L., Chilian A., Chelărescu D.E. (2013). Studies concerning heavy metals accumulation of *Carduus nutans* L. and Taraxacum officinale as potential soil bioindicator species. Rev. Chim..

[10] Kumar Sharma R., Agrawal M., Marshall F. (2007). Heavy Metal Contamination of Soil and Vegetables in Suburban Areas of Varanasi, India. Ecotoxicol. Environ. Saf..

[11] Micó C., Recatalá L., Peris M., Sánchez J. (2006). Assessing Heavy Metal Sources in Agricultural Soils of an European Mediterranean Area by Multivariate Analysis. Chemosphere.

[12] Sun C., Liu J., Wang Y., Sun L., Yu H. (2013). Multivariate and Geostatistical Analyses of the Spatial Distribution and Sources of Heavy Metals in Agricultural Soil in Dehui, Northeast China. Chemosphere.

[13] Zhuang P., McBride M.B., Xia H., Li N., Li Z. (2009). Health Risk from Heavy Metals via Consumption of Food Crops in the Vicinity of Dabaoshan Mine, South China. Sci. Total Environ..

[14] Banat K.M., Howari F.M., Al-Hamad A.A. (2005). Heavy Metals in Urban Soils of Central Jordan: Should We Worry about Their Environmental Risks?. Environ. Res..

[15] Zalidis G., Stamatiadis S., Takavakoglou V., Eskridge K., Misopolinos N. (2002). Impacts of Agricultural Practices on Soil and Water Quality in the Mediterranean Region and Proposed Assessment Methodology. Agric. Ecosyst. Environ..

[16] Jerome O.N., Pacyna J.M. (1988). Quantitative assessment of worldwide contamination of air, water and soils by trace metals. Nature.

[17] Sacchi E., Mallen L., Facchinelli A. (2001). Multivariate Statistical and GIS-Based Approach to Identify Heavy Metal Sources in Soils. Environ. Pollut..

[18] Aydinalp C., Marinova S. (2003). Distribution and Forms of Heavy Metals in Some Agricultural Soils. Pol. J. Environ. Stud..

[19] Bruemmer G.W., Gerth J., Herms U. (1986). Heavy Metal Species, Mobility and Availability in Soils. Z. Pflanz. Bodenkd..

[20] Parveen N., Ghaffar A., Shirazi S.A., Bhalli M.N. (2012). A GIS Based Assessment of Heavy Metals Contamination in Surface Soil of Urban Parks: A Case Study of Faisalabad City-Pakistan. J. Geogr. Nat. Disasters.

[21] Acosta J.A., Faz A., Martínez-Martínez S., Arocena J.M. (2011). Enrichment of Metals in Soils Subjected to Different Land Uses in a Typical Mediterranean Environment (Murcia City, Southeast Spain). Appl. Geochem..

[22] Wilcke W., Müller S., Kanchanakool N., Zech W. (1998). Urban Soil Contamination in Bangkok: Heavy Metal and Aluminium Partitioning in Topsoils. Geoderma.

[23] Li X., Lee S.I., Wong S.C., Shi W., Thornton I. (2004). The Study of Metal Contamination in Urban Soils of Hong Kong Using a GIS-Based Approach. Environ. Pollut..

[24] Papadopoulos A., Prochaska C., Papadopoulos F., Gantidis N., Metaxa E. (2007). Determination and Evaluation of Cadmium, Copper, Nickel, and Zinc in Agricultural Soils of Western Macedonia, Greece. Environ. Manag..

[25] Vavoulidou E., Avramides E.J., Papadopoulos P., Dimirkou A., Charoulis A., Konstantinidou-Doltsinis S. (2005). Copper Content in Agricultural Soils Related to Cropping Systems in Different Regions of Greece. Commun. Soil Sci. Plant Anal..

[26] Heikens A. (2006). Arsenic Contamination of Irrigation Water, Soil and Crops in Bangladesh: Risk Implications for Sustainable Agriculture and Food Safety in Asia. FAO RAP Publ..

[27] Sillanpää M. (1976). Trace Elements in Soil and Agriculture. FAO Soils Bulletin.

[28] Wuana R.A., Okieimen F.E. (2011). Heavy Metals in Contaminated Soils: A Review of Sources, Chemistry, Risks and Best Available Strategies for Remediation. ISRN Ecol..

[29] Musa D., Muazu A., Ujih U., Sabiu N., Abubakar M., Gebbe H., Chiroma Y. (2016). Bioconcentration of Heavy Metals by Wild Plants Along Holomorphic Soils in Sule-Tankarkar Local Government Area, Jigawa State, Nigeria. J. Nat. Sci. Res..

[30] Altland J. (2006). Managing Manganese Deficiency in Nursery Production of Red Maple.

[31] Llopis C., Peris M., Sánchez J., Recatalá L. (2006). Heavy Metal Content of Agricultural Soils in a Mediterranean Semiarid Area: The Segura River Valley (Alicante, Spain). Span. J. Agric. Res..

[32] Melo L.C.A., Alleoni L.R.F., Carvalho G., Azevedo R.A. (2011). Cadmium- and Barium-Toxicity Effects on Growth and Antioxidant Capacity of Soybean (*Glycine max* L.) Plants, Grown in Two Soil Types with Different Physicochemical Properties. J. Plant Nutr. Soil Sci..

[33] Lewandowski A., Zumwinkle M. Assessing the Soil System. A Soil Quality Literature Review. https://wrl.mnpals.net/islandora/object/WRLrepository%3A3001/datastream/PDF/view.

[34] Moldoveanu A.M., Hernandez-Soriano M.C. (2014). Assessment of Soil Pollution with Heavy Metals in Romania. Environmental Risk Assessment of Soil Contamination.

[35] Hamad R., Balzter H., Kolo K. (2019). Assessment of Heavy Metal Release into the Soil after Mine Clearing in Halgurd-Sakran National Park, Kurdistan, Iraq. Environ. Sci. Pollut. Res..

[36] Moor C., Lymberopoulou T., Dietrich V.J. (2001). Determination of Heavy Metals in Soils, Sediments and Geological Materials by ICP-AES and ICP-MS. Mikrochim. Acta.

[37] Shokr M.S., El Baroudy A.A., Fullen M.A., El-beshbeshy T.R., Ramadan A.R., Abd El Halim A., Guerra A.J.T., Jorge M.C.O. (2016). Spatial Distribution of Heavy Metals in the Middle Nile Delta of Egypt. Int. Soil Water Conserv. Res..

[38] Al-Taani A., Nazzal Y., Howari F., Iqbal J., Bou-Orm N., Xavier C.M., Bărbulescu A., Sharma M., Dumitriu C.S. (2021). Contamination assessment of heavy metals in soil, Liwa area, UAE. Toxics.

[39] Nazzal Y., Alarifi N., Jafri M., Kishawy H.A., Ghrefat H., Elwaheidi M., Batayneh A., Zumlot T. (2015). Multivariate Statistical Analysis of Urban Soil Contamination by Heavy Metals at Selected Industrial Locations in the Greater Toronto Area, Canada. Geol. Croat..

[40] Castrignanò A., Costantini E.A.C., Barbetti R., Sollitto D. (2009). Accounting for Extensive Topographic and Pedologic Secondary Information to Improve Soil Mapping. Catena.

[41] Reghunath R., Murthy T.R.S., Raghavan B.R. (2002). The Utility of Multivariate Statistical Techniques in Hydrogeochemical Studies: An Example from Karnataka, India. Water Res..

[42] Lu A., Wang J., Qin X., Wang K., Han P., Zhang S. (2012). Multivariate and Geostatistical Analyses of the Spatial Distribution and Origin of Heavy Metals in the Agricultural Soils in Shunyi, Beijing, China. Sci. Total Environ..

[43] Venkatramanan S., Chung S.Y., Lee S.Y., Park N. (2014). Assessment of River Water Quality via Environmentric Multivariate Statistical Tools and Water Quality Index: A Case Study of Nakdong River Basin, Korea. Carpathian J. Earth Environ. Sci..

[44] Lagacherie P., McBratney A.B., Lagacherie P., McBratney A.B., Voltz M.B.T.-D. (2006). Spatial Soil Information Systems and Spatial Soil Inference Systems: Perspectives for Digital Soil Mapping. Digital Soil Mapping.

[45] Lucà F., Buttafuoco G., Terranova O. (2018). GIS and Soil. Compr. Geogr. Inf. Syst..

[46] Das Chagas P.S.F., de Souza M.F., Dombroski J.L.D., de Junior R.S.O., de Nunes G.H.S., Pereira G.A.M., Silva T.S., Passos A.B.R., dos Santos J.B., Silva D.V. (2019). Multivariate Analysis Reveals Significant Diuron-Related Changes in the Soil Composition of Different Brazilian Regions. Sci. Rep..

[47] Theocharopoulos S.P., Petrakis P.V., Trikatsoula A. (1997). Multivariate Analysis of Soil Grid Data as a Soil Classification and Mapping Tool: The Case Study of a Homogeneous Plain in Vagia, Viotia, Greece. Geoderma.

[48] Oumenskou H., El Baghdadi M., Barakat A., Aquit M., Ennaji W., Karroum L.A., Aadraoui M. (2019). Multivariate Statistical Analysis for Spatial Evaluation of Physicochemical Properties of Agricultural Soils from Beni-Amir Irrigated Perimeter, Tadla Plain, Morocco. Geol. Ecol. Landsc..

[49] Jin Y., O’Connor D., Ok Y.S., Tsang D.C.W., Liu A., Hou D. (2019). Assessment of Sources of Heavy Metals in Soil and Dust at Children’s Playgrounds in Beijing Using GIS and Multivariate Statistical Analysis. Environ. Int..

[50] Rodrigo-Comino J., Keshavarzi A., Bagherzadeh A., Brevik E.C. (2019). The Use of Multivariate Statistical Analysis and Soil Quality Indices as Tools to Be Included in Regional Management Plans. A Case Study from the Mashhad Plain, Iran. Geogr. Res. Lett..

[51] Shriadah M.M.A. (1999). Heavy Metals in Mangrove Sediments of the United Arab Emirates Shoreline (Arabian Gulf). Water Air Soil Pollut..

[52] El Tokhi M., Amin B., Alaabed S.A. (2017). Environmental Assessment of Heavy Metals Contamination of Bottom Sediments of Oman Gulf, United Arab Emirates. J. Pollut. Eff. Control.

[53] Howari F.M. (2005). Distribution of Heavy Metal Concentrations in Surface Sediments in Dubai Creeks, United Arab Emirates. Ann. Chim..

[54] Bărbulescu A., Nazzal Y., Howari F. (2020). Assessing the groundwater quality in the Liwa area, the United Arab Emirates. Water.

[55] Mahmoud M.T., Hamouda M.A., Al Kendi R.R., Mohamed M.M. (2018). Health Risk Assessment of Household Drinking Water in a District in the UAE. Water.

[56] Jolly J., Ahmed F. (2018). Spectrophotometric Studies of Sea Water Samples of United Arab Emirates. Int. J. Adv. Sci. Eng. Technol..

[57] Samara F., Elsayed Y., Soghomonian B., Knuteson S.L. (2016). Chemical and Biological Assessment of Sediments and Water of Khalid Khor, Sharjah, United Arab Emirates. Mar. Pollut. Bull..

[58] Ajaj R., Shahin S., Kurup S., Cheruth A.-J., Salem M.A. (2018). Elemental Fingerprint of Agriculture Soils of Eastern Region of the Arabian Desert by ICP-OES with GIS Mapping. Curr. Environ. Eng..

[59] Nourzadeh M., Mahdian M.H., Malakouti M.J., Khavazi K. (2012). Investigation and Prediction Spatial Variability in Chemical Properties of Agricultural Soil Using Geostatistics. Arch. Agron. Soil Sci..

[60] Wälder K., Wälder O., Rinklebe J., Menz J. (2008). Estimation of Soil Properties with Geostatistical Methods in Floodplains. Arch. Agron. Soil Sci..

[61] Iqbal J., Nazzal Y., Howari F., Xavier C., Yousef A. (2018). Hydrochemical Processes Determining the Groundwater Quality for Irrigation Use in an Arid Environment: The Case of Liwa Aquifer, Abu Dhabi, United Arab Emirates. Groundw. Sustain. Dev..

[62] Farrant A., Ellison R., Merritt J., Merritt J.E., Newell A., Lee J., Price S.J., Leslie A., Thomas B., Farrant A.R. (2012). Geology of the Abu Dhabi 1:100,000 Map Sheet, 100–116, United Arab Emirates.

[63] Shahid S., Abdelfattah M. (2008). Soils of Abu Dhabi Emirate. Terrestrial Environment of Abu Dhabi Emirate.

[64] Shahid S.A., Taha F.K., Abdelfattah M.A. (2013). Developments in Soil Classification, Land Use Planning and Policy Implications: Innovative Thinking of Soil Inventory for Land Use Planning and Management of Land Resources.

[65] Imakumbili I.M.L. (2019). Soil Sampling and Preparation for Soil Chemical Analysis. PLoS ONE.

[66] (2007). U.S. EPA Method 3051A (SW-846): Microwave Assisted Acid Digestion of Sediments, Sludges, and Oils. https://www.epa.gov/hw-sw846/sw-846-test-method-3051a-microwave-assisted-acid-digestion-sediments-sludges-soils-and-oils.

[67] European Reference Material, Certified Reference Material ERM®-CC141. https://www.researchgate.net/publication/236902288_The_Certification_of_the_Mass_Fraction_of_the_Total_Content_and_the_Aqua_Regia_Extractable_Content_of_As_Cd_Co_Cr_Cu_Mn_Ni_Pb_and_Zn_in_Loam_Soil_Certified_Reference_Material_ERM-CC141.

[68] Jolliffe I. (2014). Principal Component Analysis.

[69] Kaiser H.F., Rice J. (1974). Little Jiffy, Mark Iv. Educ. Psychol. Meas..

[70] Cattell R.B. (1966). The Scree Test for The Number Of Factors. Multivar. Behav. Res..

[71] Gorsuch R.L. (1974). Factor Analysis.

[72] Zwick W., Velicer W. (1982). Factors Influencing Four Rules For Determining The Number Of Components to Retain. Multivar. Behav. Res..

[73] GeeksforGeeks. https://www.geeksforgeeks.org/ml-hierarchical-clustering-agglomerative-and-divisive-clustering/.

[74] Kowalska J.B., Mazurek R., Gąsiorek M., Zaleski T. (2018). Pollution indices as useful tools for the comprehensive evaluation of the degree of soil contamination—A review. Environ. Geochem. Health.

[75] Al-Hejuje M.M., Al-Saad H.T., Hussain N.A. (2018). Application of geo-accumulation index (I-geo) for assessment the sediments contamination with heavy metals at Shatt Al-Arab River-Iraq. J. Sci. Eng. Resear..

[76] Müller G. (1971). Heavy Metals in the Sediments of the Rhine: Changes since 1971. A look around. Sci. Technol..

[77] Sutherland R.A. (2000). Bed sediment-associated trace metals in an urban stream, Oahu, Hawaii. Environ. Geol..

[78] Turekian K.K., Wedepohl K.H. (1961). Distribution of the Elements in Some Major Units of the Earth’s Crust. GSA Bull..

[79] Badr N., El-Fiky A., Mostafa A., Al-Mur B. (2008). Metal Pollution Records in Core Sediments of Some Red Sea Coastal Areas, Kingdom of Saudi Arabia. Environ. Monit. Assess..

[80] Ray A.K., Tripathy S.C., Patra S., Sarma V.V. (2006). Assessment of Godavari Estuarine Mangrove Ecosystem through Trace Metal Studies. Environ. Int..

[81] Tomlinson D., Wilson J., Harris C.R., Jeffrey D.W. (1980). Problems in Assessment of Heavy Metals in Estuaries and the Formation of Pollution Index. Helgoländer Meeresunters..

[82] Selvam A.P., Priya S.L., Banerjee K., Hariharan G., Purvaja R., Ramesh R. (2012). Heavy Metal Assessment Using Geochemical and Statistical Tools in the Surface Sediments of Vembanad Lake, Southwest Coast of India. Environ. Monit. Assess..

[83] Cheng J., Shi Z., Zhu Y. (2007). Assessment and mapping of environmental quality in agricultural soils of Zhejiang Province, China. J. Environ. Sci..

[84] Gong Q., Deng J., Xiang Y., Wang Q., Yang L. (2008). Calculating pollution indices by heavy metals in ecological geochemistry assessment and a case study in parks of Beijing. J. China Univ. Geosci..

[85] Taylor S.R. (1964). Abundance of Chemical Elements in the Continental Crust: A New Table. Geochim. Cosmochim. Acta.

[86] Mason P.F. (1966). The Atlantic City ore mine: A study in economic geography. Prof. Geogr..

[87] Bowen H.J.M. (1966). Trace Elements in Biochemistry.

[88] Tóth G., Hermann T., Da Silva M.R., Montanarella L. (2016). Heavy Metals in Agricultural Soils of the European Union with Implications for Food Safety. Environ. Int..

[89] Rafati Rahimzadeh M., Rafati Rahimzadeh M., Kazemi S., Moghadamnia A.A. (2017). Cadmium toxicity and treatment: An update. Caspian J. Intern Med..

[90] Adriano D.C. (2001). Trace elements in Terrestrial Environments Biogeochemistry, Bioavailability and Risks of Metals.

[91] Spargo J., Allen T., Kariuki S. Interpreting Your Soil Test Results. https://ag.umass.edu/sites/ag.umass.edu/files/fact-sheets/pdf/spttl_2_interpreting_your_soil_test_results_0.pdf.

[92] Lenntech. https://www.lenntech.com/periodic/elements/cu.htm.

[93] Bhatti S.S., Kumar V., Singh N., Sambyal V., Singh J., Katnoria J.K., Nagpal A.K. (2016). Physico-Chemical Properties and Heavy Metal Contents of Soils and Kharif Crops of Punjab, India. Procedia Environ. Sci..

[94] US Department of Health and Human Services, Toxicological Profile for Zinc 2005. https://www.atsdr.cdc.gov/toxprofiles/tp60.pdf.

[95] Barabasz W., Albinska D., Jaskowska M., Lipiec J. (2002). Ecotoxicology of Aluminium. Pol. J. Environ. Stud..

[96] Al-Taani A., Nazzal Y., Howari F.M. (2019). Assessment of heavy metals in roadside dust along the Abu Dhabi–Al Ain National Highway, UAE. Environ. Earth Sci..

[97] Bărbulescu A., Nazzal Y. (2020). Statistical analysis of the dust storms in the United Arab Emirates. Atmosph. Res..

[98] Nazzal Y., Bărbulescu A., Howari F.M., Yousef A., Al-Taani A.A., Al Aydaroos F., Naseem M. (2019). New insight to dust storm from historical records, UAE. Arabian J. Geosci..

