# Supplementary material for: Assessment of Metals Concentrations in Soils of Abu Dhabi Emirate Using Pollution Indices and Multivariate Statistics
Source: Toxics. 2021 Apr 25;9(5):95. doi: 10.3390/toxics9050095 (PMC8146448; doi:10.3390/toxics9050095)
Supplement: Supplementary file 1 [file toxics-09-00095-s001.zip › toxics-1154314 supplementary.pdf]

Assessment of Metals Concentrations in Soils of Abu Dhabi Emirate Using Pollution Indices and Multivariate Statistics

Yousef Nazzal, Alina Bărbulescu, Fares Howari, Ahmed A. Al-Taani, Jibran Iqbal, Cijo M. Xavier, Manish Sharma and Cristian-Stefan Dumitriu

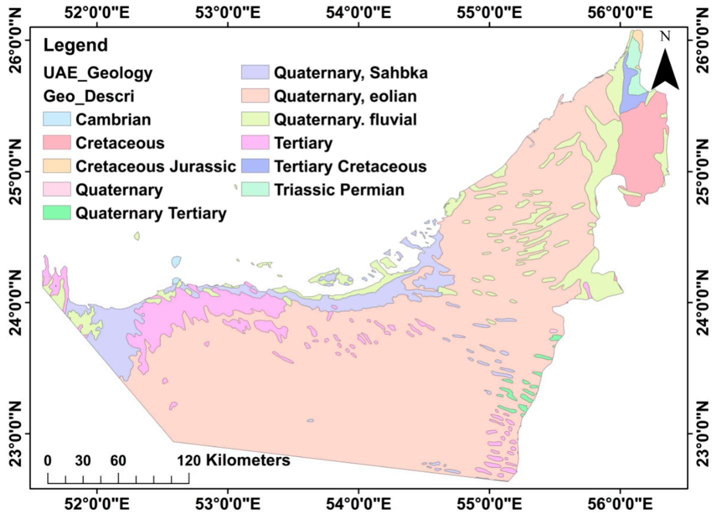

Figure S1. The geological map of the study area

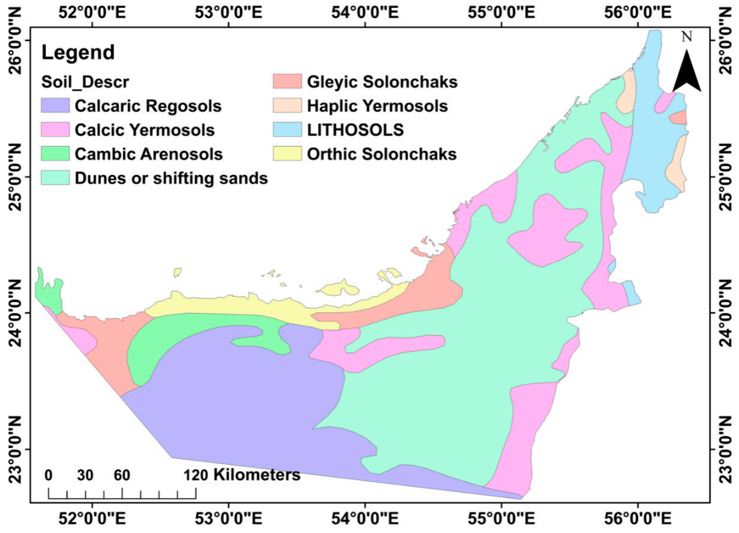

Figure S2. The soils map of the study area

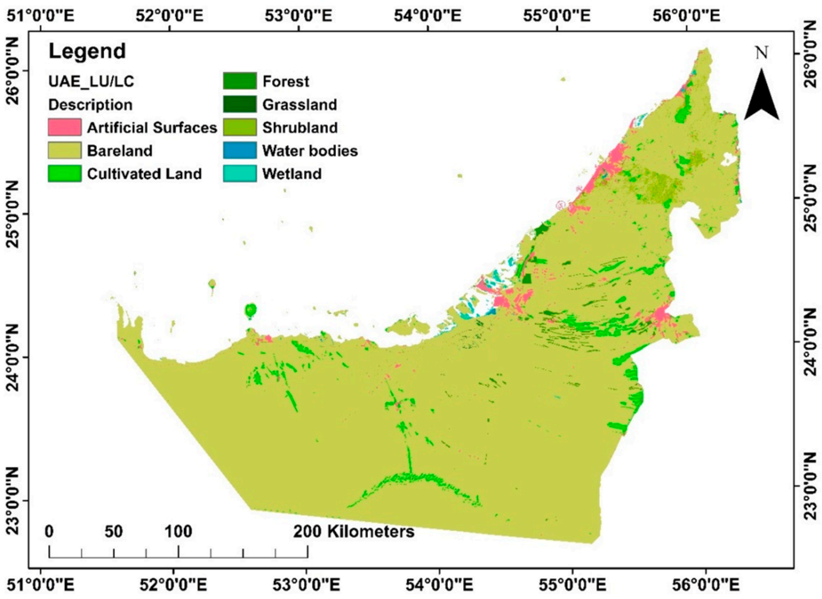

Figure S3. Land use map of the study area

	Cd	Cu	Ni	Pb	Zn	Cr	Co	Ba	Fe	Mn	As	Al	Ca	Mg	Na	Sr	K
Cd		0.260	0.043	0.081	0.142	0.079	0.010	0.336	0.143	0.082	0.148	0.303	0.107	0.054	0.060	0.213	0.095
Cu	0.260		0.059	0.092	0.218	0.035	0.362	0.300	0.025	0.003	0.000	0.346	0.073	0.492	0.210	0.448	0.000
Ni	0.043	0.059		0.000	0.000	0.000	0.000	0.000	0.100	0.000	0.000	0.000	0.000	0.000	0.001	0.001	0.066
Pb	0.081	0.092	0.000		0.000	0.000	0.000	0.000	0.074	0.000	0.000	0.001	0.000	0.002	0.097	0.002	0.117
Zn	0.142	0.218	0.000	0.000		0.000	0.000	0.000	0.471	0.000	0.000	0.017	0.000	0.008	0.005	0.012	0.001
Cr	0.079	0.035	0.000	0.000	0.000		0.000	0.000	0.073	0.000	0.000	0.000	0.000	0.010	0.000	0.007	0.000
Co	0.010	0.362	0.000	0.000	0.000	0.000		0.003	0.000	0.001	0.018	0.019	0.000	0.000	0.000	0.051	0.436
Ba	0.336	0.300	0.000	0.000	0.000	0.000	0.003		0.336	0.000	0.000	0.011	0.000	0.007	0.091	0.014	0.111
Fe	0.143	0.025	0.100	0.074	0.471	0.073	0.000	0.336		0.045	0.015	0.011	0.498	0.000	0.395	0.218	0.001
Mn	0.082	0.003	0.000	0.000	0.000	0.000	0.001	0.000	0.045		0.000	0.000	0.000	0.006	0.002	0.002	0.000
As	0.148	0.000	0.000	0.000	0.000	0.000	0.018	0.000	0.015	0.000		0.000	0.000	0.031	0.001	0.033	0.000
Al	0.303	0.346	0.000	0.001	0.017	0.000	0.019	0.011	0.011	0.000	0.000		0.000	0.001	0.347	0.001	0.211
Ca	0.107	0.073	0.000	0.000	0.000	0.000	0.000	0.000	0.498	0.000	0.000	0.000		0.000	0.005	0.000	0.000
Mg	0.054	0.492	0.000	0.002	0.008	0.010	0.000	0.007	0.000	0.006	0.031	0.001	0.000		0.001	0.002	0.372
Na	0.060	0.210	0.001	0.097	0.005	0.000	0.000	0.091	0.395	0.002	0.001	0.347	0.005	0.001		0.045	0.001
Sr	0.213	0.448	0.001	0.002	0.012	0.007	0.051	0.014	0.218	0.002	0.033	0.001	0.000	0.002	0.045		0.342
K	0.095	0.000	0.066	0.117	0.001	0.000	0.436	0.111	0.001	0.000	0.000	0.211	0.000	0.372	0.001	0.342	

Figure S4. p-values corresponding to the correlation coefficients between the metals concentration

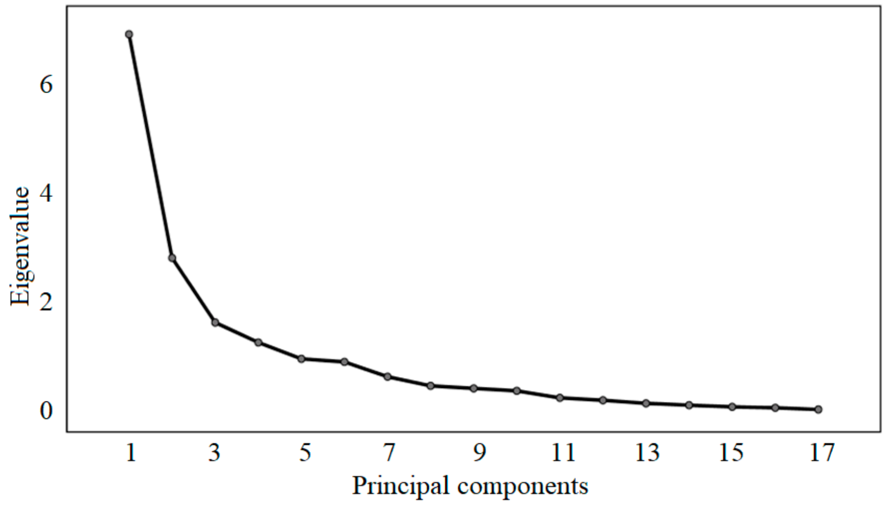

Figure S5. The scree plot

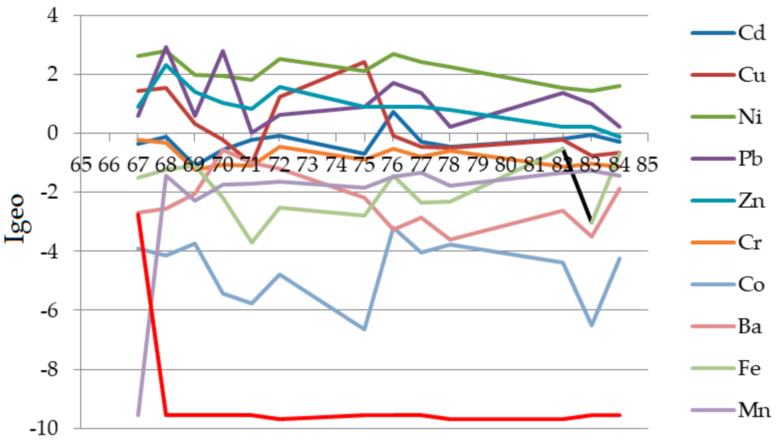

Figure S6. Igeo for the sites from cluster 3

Table S1. Geo-accumulation index (Igeo) for contamination levels, adapted from Muller [76]

Igeo class	Igeo value	Contamination level
0	Igeo ≤ 0	Uncontaminated
1	0 < Igeo < 1	Uncontaminated/Moderately contaminated
2	1 ≤ Igeo < 2	Moderately contaminated
3	2 ≤ Igeo < 3	Moderately/Strongly contaminated
4	3 ≤ Igeo < 4	Strongly contaminated
5	4 ≤ Igeo < 5	Strongly/Extremely contaminated
6	Igeo ≥5	Extremely contaminated

Table S2. Average crustal abundance and average world soils concentrations [78,85-87].

Element	Average crustal abundances (mg/kg) [78,85,86]	Soils average (mg/kg)[86,87]
Cd	0.2	0.5
Cu	55	20
Ni	75	40
Pb	20	10
Zn	75	50
Cr	100	100
Co	25	15
Ba	415	500
Fe	50000	3800
Mn	20900	850
As	1.8	5
Al	81300	5000
Ca	36300	13700
Mg	20900	500
Na	28300	6300
Sr	375	300
K	25900	14000

Table S3. Results of the Principal Component Analysis – factors' importance.

PC	Initial eigenvalues			Extracted sums of squared loadings		
	Total	Variance (%)	Cumulative Variance (%)	Total	Variance (%)	Cumulative (%)
1	6.873	40.431	40.431	6.873	40.431	40.431
2	2.789	16.406	56.836	2.789	16.406	56.836
3	1.610	9.470	66.306	1.610	9.470	66.306
4	1.246	7.328	73.634	1.246	7.328	73.634
5	0.947	5.568	79.202			
6	0.891	5.239	84.441			
7	0.620	3.646	88.088			
8	0.454	2.669	90.757			
9	0.407	2.395	93.152			
10	0.362	2.131	95.283			
11	0.234	1.378	96.661			
12	0.190	1.118	97.779			
13	0.134	0.786	98.564			

14	0.100	0.590	99.154
15	0.070	0.409	99.563
16	0.053	0.310	99.873
17	0.022	0.127	100.000

Table S4. Principal components.

Element	PC1	PC2	PC3	PC4
Mn	**0.905**	−0.340	0.006	-0.026
Cr	**0.872**	−0.267	−0.023	0.083
Ca	**0.869**	−0.037	−0.070	−0.323
Ni	**0.861**	0.283	−0.202	0.176
As	**0.810**	−0.477	0.119	−0.044
Zn	**0.767**	0.035	−0.295	0.285
Pb	**0.729**	0.258	−0.390	0.114
Ba	**0.668**	0.139	−0.403	0.103
Co	**0.564**	**0.562**	0.378	0.206
Na	0.427	0.003	0.413	0.407
Fe	0.062	**0.800**	0.328	−0.127
K	0.548	**−0.694**	0.307	0.055
Mg	0.508	**0.608**	0.480	−0.075
Cu	−0.152	**0.566**	**−0.524**	0.239
Cd	0.230	0.101	0.348	0.207
Al	0.480	0.188	−0.034	**−0.616**
Sr	0.458	0.182	−0.070	**−0.532**

Rotation Method: Oblimin with Kaiser Normalization.

Table S5. Rotated factor loading matrix.

Element	PC1	PC2	PC3	PC4
Mn	**0.606**	0.110	**0.696**	0.268
Cr	**0.645**	0.149	**0.607**	0.176
Ca	**0.559**	0.137	0.421	**0.599**
Ni	**0.842**	0.352	0.097	0.225
As	0.460	0.069	**0.800**	0.202
Zn	**0.833**	0.145	0.206	0.036
Pb	**0.828**	0.145	−0.021	0.232
Ba	0.774	0.045	0.039	0.190
Co	0.337	**0.827**	−0.025	0.142
Na	0.213	**0.545**	0.356	−0.220
Fe	−0.084	**0.670**	−0.460	0.318
K	0.154	0.026	**0.925**	−0.026
Mg	0.147	**0.833**	−0.038	0.383
Cu	0.345	−0.041	**−0.734**	−0.121
Cd	0.045	0.435	0.168	−0.077
Al	0.185	0.078	0.083	**0.773**
Sr	0.212	0.065	0.065	**0.695**
